# The Electroencephalographic Characterization of Hypsarrhythmia in Older Pediatric Population With Epilepsy Using Computer-Added Quantitative Methods

**DOI:** 10.7759/cureus.34586

**Published:** 2023-02-03

**Authors:** Kamlesh Jha, Tribhuwan Kumar, Md. Zabihullah, Yogesh Kumar, Rajesh Kumar, Abhilasha Mishra

**Affiliations:** 1 Physiology, All India Institute of Medical Sciences, Patna, Patna, IND; 2 Obstetrics and Gynaecology, Swami Vivekanand Hospital, New Delhi, IND

**Keywords:** otahara syndrome, west syndrome, krammer scoring system, electroencephalography, pediatric seizure, infantile spasm, qeeg, power spectral density, hypsarrhythmia

## Abstract

Background

Hypsarrhythmia is a classical multifocal electroencephalographic finding in patients of infantile spasm and related epileptic syndromes of early childhood including West syndrome and Otahara syndrome. It usually presents in early infancy and persists up to the age of two years, after which it usually resolves. The persistence of hypsarrhythmia beyond the age of two years has rarely been reported in the literature. The present study is an attempt to investigate and compare the origin and activation pattern of epileptic activity between the subjects aged 3-10 years with and without hypsarrythmia.

Material and methods

Forty-one patients in the age group of 3-10 years with features suggestive of seizure have been studied for quantitative electroencephalographic characteristics after dividing into hypsarrythmic and normal seizure patterns.

Result

The power spectral density (PSD) of 15 patients with hypsarrhythmia showed a significantly predominant delta frequency in quantitative electrography (qEEG) in comparison to the seizure subjects with normal electroencephalography (EEG) patterns. The amplitude progression analysis of both groups showed that the origin of focus of the hypsarrhythmic pattern is from the occipital region while no such pattern has been noticed in the control group.

Discussion and conclusion

Hypsarrythmia is known to show multifocal origin. Predominant occipital origin in older age group subjects distinguishes the condition from classical hypsarrythmia of early childhood. The occipital origin may be indicative of persistent immaturity of the thalamocortical synaptic pathway.

## Introduction

Electroencephalography (EEG) is an invaluable tool in the diagnosis and prognosis of seizures. Despite much advancement in electrodiagnostic techniques and wide acceptance in seizure diagnosis, EEG is yet to attain high sensitivity and specificity in seizure diagnostic potential [1]. Computer-added quantitative EEG (qEEG), which has evolved recently, can add value to the potential of EEG in seizure diagnostics [2]. Pediatric seizures place further diagnostic and prognostic challenges owing to inconspicuous signs and symptoms and atypical EEG features associated with them. Hypsarrhythmia is one of the characteristic EEG findings in infantile spasms, a type of atypical pediatric seizure. Persistence of hypsarrhythmia beyond the age of three years is rarely reported in the literature [3]. Its presence at a later age may be a unique EEG feature indicative of some condition other than infantile spasm in the particular age group.

Infantile spasm is a unique pediatric seizure described by various names including Salaam attack, Salaam tick, Salaam convulsion, propulsive petit mal, flexon spasm, nodding spasm, and lightening spasm [4]. For ages, the condition inconspicuously represented pediatric seizure in general. After the advent of sophisticated electrodiagnostic tools like EEG and keen observations by clinicians, it has been recognized as a distinct entity associated with pediatric seizures.

Hypsarrhythmia is an EEG finding, characteristically present in infantile spasm and West syndrome [5]. The word "Hypsarrhythmia" is derived from "Hypsi" meaning high or mountainous and "Arrhythmia" meaning lack of rhythm. It is an EEG finding characterized by multifocal spike activity over a high voltage chaotic slow wave background [6].

This classical presentation, however, is not a very common finding during EEG recording. Instead, various other forms of hypsarrhythmia are more likely to be found in laboratory settings. These patterns are commonly known as modified hypsarrhythmia. These modified patterns can be seen as hemispheric synchronization, hypsarrhythmia with a consistent focus, hypsarrhythmia with voltage attenuation, or hypsarrhythmia with few spikes or sharp waves [7].

Various scoring systems have been used to define and assess the severity of hypsarrhythmia, in which the Krammer scoring system is one of the most frequently cited in the literature [8]. In this scoring system, items scored include disorganization, delta activity, amplitude, and spike activity with scores from 0 to 3 each. In addition, a score of 1 each was given to any of the following additional patterns: electro-decremental response, burst suppression, absence of sleep pattern, and relative normalization. The maximum possible score in this scoring system is 16, which indicates the most severe type of presentation including completely disorganized high voltage delta activity interspersed with spike waves, burst suppression pattern, and electro-decremental pattern throughout the record. Hypsarrhythmia usually develops in early infancy and persists till the age of two years. After that, it evolves into various other patterns due to its dynamic nature. The disappearance of hypsarrhythmia [9] may occur due to the restructuring of a neuronal network [10] during the evolution of West syndrome or during the natural maturation of the frontostriatal network with more sufficient control of the frontal cortex over the striatopallidal system [11].

Finding hypsarrhythmia in children beyond the age of three years is unusual. If it is present beyond this age, it means that the tracts involved in the production of these high voltage slow waves are still immature or the frontal control has not been developed yet. A case study has proposed that there might be involvement of both cortical structure as well as subcortical structures in the development of hypsarrhythmia as shown by positron emission tomography (PET) and single photon emission computerized tomography (SPECT) studies but the exact pathway could not be specified by these studies [12]. Further, the chaotic EEG pattern gives little information clinically regarding the focus of the electrical rhythm abnormalities.

Assessment of hemodynamic responses by simultaneous EEG and functional magnetic resonance imaging (fMRI) has revealed that high-voltage slow waves are produced from subcortical structures including the thalamus, hippocampus, and brainstem nuclei [13].

The present study is an attempt to investigate and compare the pattern of activation of the seizure activity among subjects with and without hypsarrythmia presenting with epileptic spasms. It highlights the qEEG features present among toddlers presenting with a pediatric seizure. Moreover, the study attempts to find out the focus of origin of the electrical abnormality through quantitative analysis methods.

## Materials and methods

Patient selection

Forty-one patients were included in the study from the cohort of pediatric patients referred to the Clinical Neurophysiology Lab of the Department of Physiology, All India Institute of Medical Sciences, Patna, Bihar, India, for EEG evaluation of the presenting symptoms suggestive of seizure following the pre-defined inclusion and exclusion criteria. The inclusion criteria included the age range of 3-10 years with EEG findings confirming the hypsarrhythmia (for the hypsarrhythmia group/Group 1) or definite clinical history of occurrence of seizure episodes recently (within the past two months) without definite seizure-associated EEG findings (for the control group/Group 2). Patients with a history of head trauma, associated mental retardation, and known electrolyte abnormality have been excluded from the study. Written informed consent was obtained from the parents/guardians of every patient who took part in the study. Ethical approval was obtained from the Institutional Ethical Committee of All India Institute of Medical Sciences, Patna, India (Approval number: AIIMS/Pat/IEC/2018/05/257).

EEG data acquisition

The EEG of each patient was done in the neurophysiology lab of the Physiology Department. Silver-Silver Chloride (Ag-AgCl) disc-type electrodes of 5-7 mm diameter were placed with due precautions using a 10-20 international electrode placement system. An electrode impedance of less than 5 Kohm was achieved for all the electrodes. EEG signal was acquired on 32 channel EEG system (Superspec 32; Recorders & Medicare Systems Pvt. Ltd., Panchkula, Haryana, India) at a sampling frequency of 256 Hz using low (0.5 Hz), high (70 Hz), and notch (50 Hz) pass filters.

Based on the visual clinical EEG characteristics, the subjects were divided into two groups. The subjects having EEG patterns suggestive of hypsarrhythmia were kept in Group 1 or the test group, and the subjects having other kinds of seizures but with a normal EEG, were kept in Group 2 or the control group. A total of 15 patients were included in Group 1 and 26 patients were included in Group 2.

The qEEG feature studies included disorganization pattern, delta activity, amplitude of the various waves and spike activity, electro-decremental response, burst suppression, etc. The Kramer scoring system was used for scoring the hypsarrhythmia to get a quantitative score for the subjects. Two different neurophysiologists worked independently to score each EEG to avoid any subjective bias in the scoring. Scores greater than 9 were classified as hypsarrhythmia.

Fast Fourier transformation (FFT) was done on each recording by taking a one-second epoch from each group. For the test group, two segments were taken, one with the finding of hypsarrhythmia (high voltage slow waves) and the second with normal voltage and frequency waves.

EEG signal processing

Before quantitative analysis, Various artifacts like, eye blinks, muscle movement artifacts, ECG artifacts, etc. were identified visually as well as by using a computer-based independent component analysis (ICA) technique, and respective stretches were excluded from the analyzed part [14]. Then FFT was done on a one-second epoch data of EEG in patients of both groups. Further, Group 1 patients were analyzed again in two epochs, one having high amplitude discharges, i.e. hypsarrythmic segment, and the second having a normal amplitude segment. The progression was studied qualitatively by visual inspection. FFT was done in the right temporo-occipital (T4-O2) and left temporo-occipital (T3-O1) montages where the background activity was more prominent.

FFT

FFT is done to convert the time domain data into the frequency domain data so that the frequency characteristics can be understood in a better way. FFT transforms were used to transform the time domain EEG data to frequency domain data with its constituent frequencies spectrum, i.e. delta (0.5-3.9 Hz, theta (4-7.9 Hz), low alpha (8-10.9 Hz), high alpha (11-12.9 Hz), and beta (13-30 Hz) waves. The power spectral density of individual frequency bands was studied in the individual epochs to study the strength of specific frequencies in the region under study.

## Results

Table 1 shows the demographic and clinical criteria used for both groups in the study.

**Table 1:** Demographic and clinical characteristics of Group 1 (test group) and Group 2 (control group) subjects. GC: generalized convulsions; FC: focal convulsions; BS: burst suppression pattern; CH: classical hypsarrhythmic pattern; M: male; F: female; n: number of participants; EEG: electroencephalography

	Age of symptom onset			Age of presentation			Gender			Clinical presentation	EEG characteristics
	Mean age (years)	Age range (years)		Mean age (years)	Age range (years)		Male n (%)	Female n (%)			
Group 1	2.2	0-6		4.8	3-10		6 (40)	9 (60)		GC- 73.3%	CH- 53.3%
										FC- 6.7%	BS- 46.7%
										Others- 20%	
Group 2	2.32		0-5	4.65		3-10	17 (65.4)		9 (34.6)	GC- 61.6%	Normal EEG findings
										FC- 15.4%	
										Others- 23%	

**Figure 1:**
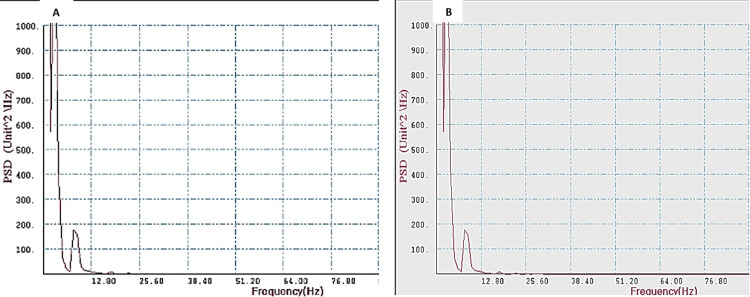
FFT for T3-O1 (A) and T4-O2 (B) in Group 1 (test group) showing PSD in various frequency ranges. PSD: power spectral density; FFT: fast Fourier transformation

**Figure 2:**
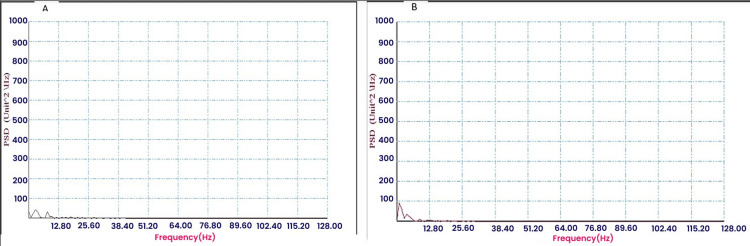
FFT for T3-O1 (A) and T4O2 (B) in Group 2 (control group) showing PSD in various frequency ranges. PSD: power spectral density; FFT: fast Fourier transformation

Figures 1-2 show the frequency plots for the FFT of T4-O2 and T3-O1 channels of Group 1 and Group 2 subjects.

The data have been tested for normality of distribution using box plot and Q-Q plot. Tests for skewness and kurtosis were also done. As the data was normally distributed, parametric tests were employed for data analysis. Variance was assessed with Fischer Test (F test). Depending on equal and unequal variance, Welch correction was done accordingly. The normal segments of Group 2 patients were compared with the normal and abnormal seizure pattern EEG signals of Group 1 patients using student’s t test.

Owing to unequal variance, Welch correction was done for the delta spectrum frequency data of Group 1 and Group 2 subjects. Both groups were compared using unpaired student’s t-test for T4-O2 and T3-O1 channels, respectively, as represented in Tables 2-3.

**Table 2:** Comparison of one-second PSD values of different frequencies in Group 2 vs hypsarrhythmic segment (Group 1) in channel T4-O2 SEM: standard error of mean; n: sample size; NS: not significant; df: degree of freedom; PSD: power spectral density

Frequency bands	Mean ± SEM of Group 2 (n=26)	Mean ± SEM of Group 1 (abnormal part) (n=15)	95% CI	Welch corrected t, df	F-value	P-value	Statistical significance
Delta (0.5-4hz)	1024 ± 251	4362 ± 588.6	2000 to 4676	t=5.216; df=19.2	3.174	<0.0001	Signifant
Theta (4-8hz)	228.9 ± 68.35	588.9 ± 213.2	-112.4 to 832.5	t=1.608; df=16.93	5.611	0.1262	NS
Low Alpha (8-12hz)	99.71 ± 24.9	68.79 ± 18.9	-94.16 to 32.31	t=0.9893; df=38.99	3.011	0.3286	NS
High Alpha (12-16hz)	4.975 ± 1.012	5.001 ± 1.043	-3.114 to 3.168	--	1.634	0.9864	NS

**Table 3:** Comparison of one-second PSD values of different frequencies in Group 2 vs hypsarrhythmic segment (Group 1) in channel T3-O1 SEM: standard error of mean; n: sample size; NS: not significant; df: degree of freedom; PSD: power spectral density

Frequency bands	Mean ± SEM of Group 2 (n=26)	Mean ± SEM of Group 1 (abnormal segment) (n=15)	95% confidence interval	Welch-corrected t, df	F-value	P-value	Statistical significance
Delta	1288 ± 466.2	3757 ± 719.8	809.1 to 4131	--	1.375	0.0046	Significant
Theta	165.7 ± 31.66	768.4 ± 305.2	-54.19 to 1260	t=1.964; df=14.3	53.62	0.0693	NS
Low Alpha	71.03 ± 12.88	86.33 ± 43.74	-81.14 to 111.7	t=0.3357; df=16.46	6.658	0.7414	NS
High Alpha	5.243 ± 1.007	3.475 ± 0.6144	-4.156 to 0.6202	t=1.499; df=37.74	4.654	0.1422	NS

**Figure 3:**
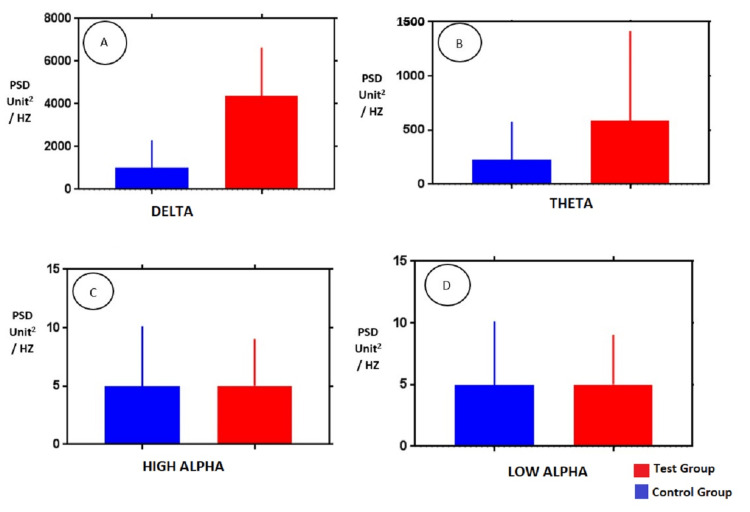
Comparison of PSD values of (A) delta, (B) theta, (C) high alpha, and (D) low alpha frequency spectrum EEG activities in Group 1 and Group 2 subjects in channel T4-O2 PSD: power spectral density; EEG: electroencephalography

**Figure 4:**
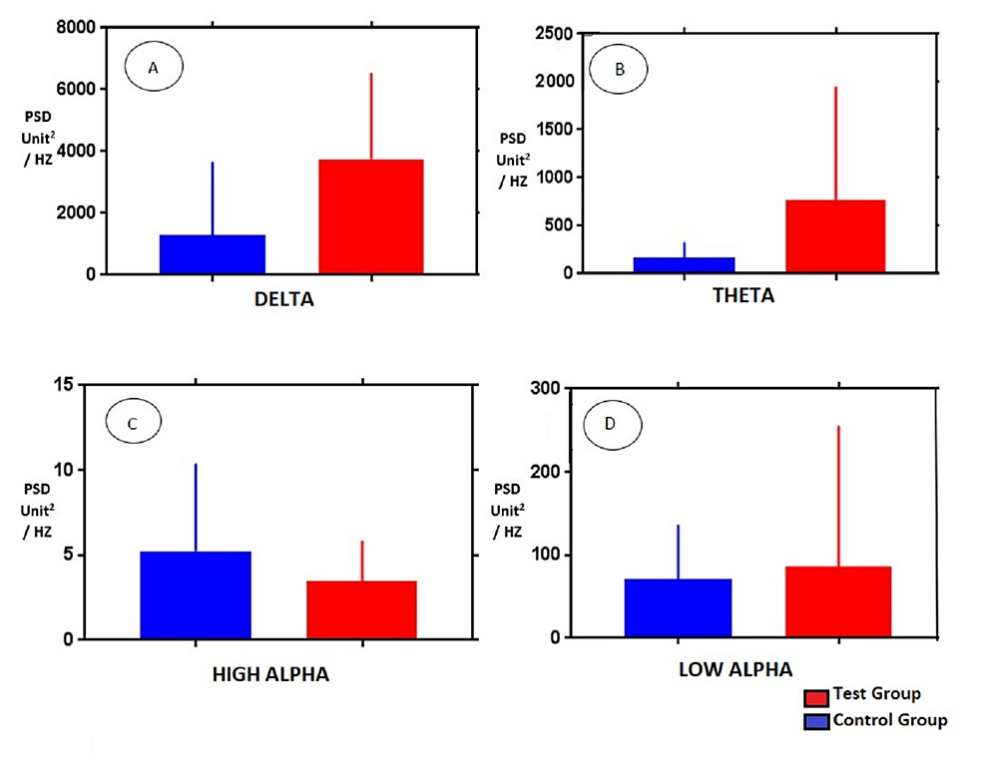
Comparison of PSD values of (A) delta, (B) theta, (C) high alpha, and (D) low alpha frequency spectrum EEG activities in Group 1 and Group 2 subjects in channel T3-O1 PSD: power spectral density; EEG: electroencephalography

Figures 3-4 represent the bar graph constructed to display the difference between the PSD of both groups on two sides in various frequency bands.

After comparing other frequencies of Group 1 and Group 2 like theta, low alpha, and high alpha, no significant difference was found though the PSD values for both groups showed less intergroup difference as the spectrum of frequency progressed from lower to a higher frequency.

**Figure 5:**
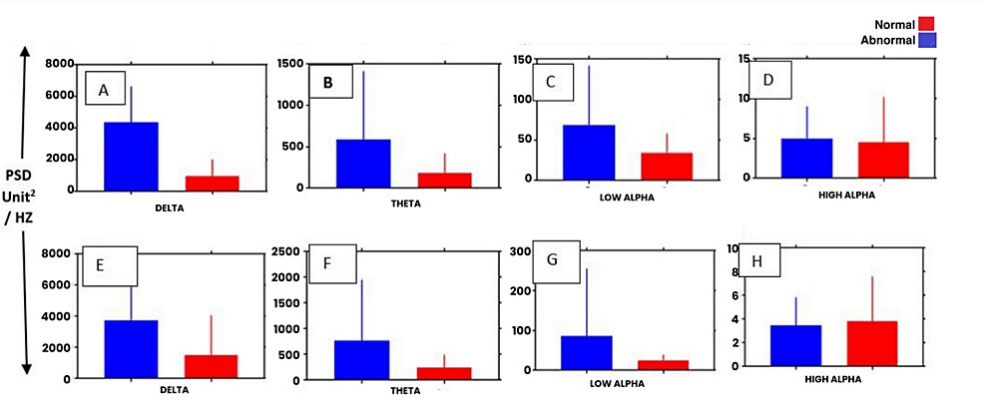
Comparison of the PSD values of various frequency spectrums of abnormal and normal segments of Group 1 subjects in channels T4-O2 (A to D) and T3-O1 (E to H). PSD: power spectral density

To further explore the frequency characteristics of Group 1 (test group) EEG record, the epochs with hypsarrythmic patterns were compared with the epochs with apparently normal-looking patterns. The PSD values from the left side mirrored the right-sided values with a significant difference found only in the delta frequency range. When the hypsarrythmic segment of temporo-occipital montage PSD data of both sides were compared with that of the non-hypsarrythmic segments of the same montage, the PSD of delta frequency was significantly higher than that of non-hypsarrythmic segments. Other frequency bands showed non-significant differences in their power spectrum (Tables 4, 5) and insignificant intergroup differences as shown in Figure 5.

**Table 4:** Comparison of one-second PSD values of different frequencies in hypsarrhythmic segment vs normal segment in channel T4-O2 of Group 1. SEM: standard error of mean; n: sample size; NS: not significant; df: degree of freedom; PSD: power spectral density

Frequency bands	Mean ± SEM of abnormal segment (n=15)	Mean ± SEM of normal segment (n=15)	95% confidence interval	Welch-corrected t, df	F-value	P-value	Statistical significance
Delta	4362 ± 588.6	974.5 ± 274.1	-4743 to -2032	t=5.217 df=19.8	4.611	<0.0001	Significant
Theta	588.9 ± 213.2	187.2 ± 60.23	-870.8 to 67.35	t=1.814 df=16.22	12.53	0.0883	NS
Low alpha	68.79 ± 18.9	34.49 ± 6.152	-76.24 to 7.647	t=1.726 df=16.93	9.434	0.1026	NS
High alpha	5.001 ± 1.043	4.545 ± 1.463	-4.137 to 3.224	--	1.969	0.8013	NS

**Table 5:** Comparison of one-second PSD values of different frequencies in hypsarrhythmic segment vs normal segment in channel T3-O1 of Group 1. SEM: standard error of mean; n: sample size; NS: not significant; df: degree of freedom; PSD: power spectral density

FREQUENCIES	Mean ± SEM of abnormal segment (n=15)	Mean ± SEM of normal segment (n=15)	95% confidence interval	Welch-corrected t, df	F-value	P-value	Statistical significance
Delta	3757 ± 719.8	1504 ± 663.9	-4259 to -247.7	--	1.175	0.0290	Significant
Theta	768.4 ± 305.2	246 ± 63.94	-1186 to 141.4	t=1.675 df=15.23	22.79	0.1143	NS
Low alpha	86.33 ± 43.74	25.3 ± 3.731	-155.1 to 33	t=1.8; df=15.8	13.75	0.1859	NS
High alpha	3.475 ± 0.6144	3.818 ± 0.9821	-2.03 to 2.715	--	2.555	0.7697	NS

**Figure 6:**
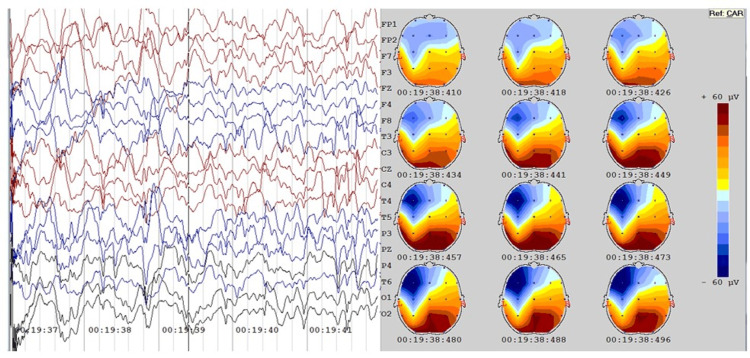
Group 1 (test group) progressive amplitude analysis in O1 channel at the time scale 19:38:410 to 19:38:496.

Progressive amplitude analysis of each patient was done to find out the focus of origin of these high amplitude discharges (Figure 6). In most of the Group 1 (test group) patients (n=11), first high amplitude discharge was noticed in the occipital area, then it spread to other adjacent areas. However, in some patients (n=4), occipitotemporal areas have also been noticed as the focus of origin. In Group 2 (control group) patients, no definite pattern could be ascertained.

## Discussion

Typical hypsarrhythmic pattern in the EEG of children presenting with seizure symptoms is rare beyond the age of three years as per the various available literature. The persistence of classical hypsarrythmic patterns in older age groups has been found to be associated with a spectrum of residual neuro-developmental abnormalities. To date, most of the observation in the area is based upon subjective clinical observations reported mostly by clinicians across the world. The current study has attempted to characterize the EEG pattern in the most objective manner with the use of qEEG signal processing methods. Objective characterization of the condition may pave a way for further research in the direction and open the avenue for objectivized subject-specific medical intervention and preventive strategies.

Most of the subjects in the study with hypsarrythmic patterns have shown epochs of completely chaotic patterns interspersed with a relatively organized EEG pattern with slow wave background. This was in congruence with the classical hypsarrythmic patterns where the whole of the record shows mostly chaotic background with diffused slowing and high voltage random spike discharges. The background shows an increase in the power of the delta frequency band on both sides, mostly located occipitally.

As discussed above, infantile spasm or its typical EEG correlate, the hypsarrythmia, develops in early infancy and persists up to two years of age. After that, it gets converted into either other forms of epilepsy or resolves completely with normal EEG findings. The present study indicates that the prevalence of seizures mimicking infantile spasm or its EEG equivalent is probably more than the reported prevalence, especially in the countries like India where record keeping for health is still in its infancy. In a long-term follow-up study of infantile spasm by Gibbs et al., out of 237 patients, 13 continued to have persistent hypsarrhythmic patterns beyond the age of three years [15]. Some of them were even of the age of 5-6 years.

Kulandaivel reported in his two-year follow-up study on different types of seizures that hypsarrhythmia persists beyond the age of three years [3]. Further, he added that children having hypsarrhythmia at higher ages frequently have associated severe structural abnormalities like Chiari malformation, and developmental and mitochondrial anomalies.

In yet another long-term follow-up study among West syndrome subjects, 49% died by the age of 19 years with pneumonia being the primary cause of their death. Thus, the condition not only affects the quality of life but is also responsible for mortality [16].

A study by Burroughs et al. attempted qEEG analysis to compare the coherence and spectral power of different frequencies [17]. In agreement with the present study, they found that there is increased spectral power in delta frequencies as compared to other frequencies. They also found that children with infantile spasm had marked increases in delta/theta, delta/beta, delta/high beta, theta/high beta, alpha/beta, alpha/high beta, and beta/high beta and marked decreases in the delta/alpha, theta/alpha, and theta/beta ratios. They also found that short-distance inter-electrode coherence was decreased in frontal and anterior temporal regions while longer-distant inter-electrode coherence was increased. They also concluded that these EEG findings were associated with cognitive impairments [17].

Earlier studies to delineate the origin of the ictal waves in hypsarrythmia had suggested that high amplitude waves originate from the occipital region and later spreads to other cortical regions. It has also been suggested that although high-voltage slow waves come from subcortical structures, hypsarrhythmia takes its origin from the cortex only, especially from the posterior cortex [18]. In our study, the progressive amplitude analysis at different time intervals in both groups suggests that hypsarrhythmic thalamocortical signals originate from the occipital region and later spreads to other regions to make it generalized. The study findings are further supported by the studies done by Wenzel [19], Guzzetta et al. [20], and Metsähonkala et al., [21]. They suggested that the pathogenesis of infantile spasm is taking place from the posterior cortex. They assessed visual functions by different clinical, behavioral, and electrophysiologic tests and found that visual abnormalities such as poor visual responsiveness, abnormal visual evoked potentials, and deficits in fixation shift were already present in these patients. These findings were supported by various other researchers including Linuma et al., [22] and Koo et al. [23], who demonstrated that structural and functional occipital lesions are the probable risk factors for infantile spasms.

The pathophysiology of the origin of the train of abnormal electrical activities from the occipital region may be explained by the focal posterior cortical hypometabolism, which is a commonly reported finding in the large body of literature on West syndrome [24,25].

It has even been hypothesized that the primary generator of infantile spasms would be cortical, particularly the posterior cortex. In agreement with the above findings, the present study also found the slow wave activity taking origin from the occipital area and then spreading to the adjacent part.

## Conclusions

Epileptic spasm is a common clinical presentation among children under two to three years of age. Its persistence beyond the age of three years is a rarity but has been reported in the medical literature. The present study attempted to investigate the origin of the hypsarrythmic EEG pattern among the subjects presenting with epileptic spasms and compare the pattern of activation between the subjects with and without hypsarrythmia. Findings of the study suggest that the origin of the hypsarrythmic activities was occipital in older children whereas in the younger children, it appeared to be multifocal. Secondly, the persistence of high delta power and preponderance of other slow waves were other novel findings in the elder children as compared to the younger children.

The potential of the classical hypsarrythmic pattern to indicate or associate with the underlying neurodevelopmental pathology may be utilized with further studies in other neurodevelopmental conditions to make the diagnostic exercise more objective. By analyzing progressive amplitudes at the point of origin of high voltage bands, we have found that the focal origin of hypsarrhythmia is probably in the occipital region. It is from this region that the activity apparently spreads to another region. Further research in this domain is needed to address the management part of the condition by finding specific neuronal networks involved in the persistence or new development of this pattern.
